# Commencement

**Published:** 1884-12

**Authors:** 


### Commencement.

The annual Commencement exercises of the Dental Department of the University of California, were held Nov. 11, at 8 o'clock P.M.

The following gentlemen having fulfilled the requirements of the institution, were presented for graduation: George Washington Cool, John Michael Dunn, Archibald Duncan Gleaves, Robert Nicol, Gustav Carl Reitzke, William Henry Simmons, Emer Joseph Welden.

